# Breast tumour volume and blood flow measured by MRI after one cycle of epirubicin and cyclophosphamide-based neoadjuvant chemotherapy as predictors of pathological response

**DOI:** 10.1259/bjr.20201396

**Published:** 2021-06-09

**Authors:** William Stevens, Isabelle M Farrow, Leonidas Georgiou, Andrew M Hanby, Timothy J Perren, Laura M Windel, Daniel J Wilson, Nisha Sharma, David Dodwell, Thomas A Hughes, Barbara JG Dall, David L Buckley

**Affiliations:** 1 Biomedical Imaging, University of Leeds, Leeds, UK; 2 Department of Medical Physics, German Oncology Center, Limassol, Cyprus; 3 School of Medicine, University of Leeds, Leeds, UK; 4 Dept of Medical Physics and Engineering, Leeds Teaching Hospitals NHS Trust, Leeds, UK; 5 Department of Radiology, Leeds Teaching Hospitals NHS Trust, Leeds, UK; 6 Institute of Oncology, St. James’s University Hospital, Leeds, UK; 7 Nuffield Department of Population Health, University of Oxford, Oxford, UK

## Abstract

**Objectives:**

Better markers of early response to neoadjuvant chemotherapy (NACT) in patients with breast cancer are required to enable the timely identification of non-responders and reduce unnecessary treatment side-effects. Early functional imaging may better predict response to treatment than conventional measures of tumour size. The purpose of this study was to test the hypothesis that the change in tumour blood flow after one cycle of NACT would predict pathological response.

**Methods:**

In this prospective cohort study, dynamic contrast-enhanced MRI was performed in 35 females with breast cancer before and after one cycle of epirubicin and cyclophosphamide-based NACT (EC90). Estimates of tumour blood flow and tumour volume were compared with pathological response obtained at surgery following completion of NACT.

**Results:**

Tumour blood flow at baseline (mean ± SD; 0.32 ± 0.17 ml/min/ml) reduced slightly after one cycle of NACT (0.28 ± 0.18 ml/min/ml). Following treatment 15 patients were identified as pathological responders and 20 as non-responders. There were no relationships found between tumour blood flow and pathological response. Conversely, tumour volume was found to be a good predictor of pathological response (smaller tumours did better) at both baseline (area under the receiver operating characteristic curve 0.80) and after one cycle of NACT (area under the receiver operating characteristic curve 0.81).

**Conclusion & advances in knowledge:**

The change in breast tumour blood flow following one cycle of EC90 did not predict pathological response. Tumour volume may be a better early marker of response with such agents.

## Introduction

Neoadjuvant chemotherapy (NACT) offers an increased rate of breast conserving surgery, can downsize advanced breast tumours to allow surgery and with the addition of human epidermal growth factor receptor 2 (HER2)-targeted therapy (to NACT or adjuvant chemotherapy) can improve survival in HER2-positive breast cancer.^1^ However, NACT comes at the cost of significant side-effects and it is therefore important to identify non-responders to NACT as early as possible. Typically, a change in tumour size is demonstrated after two or more cycles of NACT^2^ but several studies have examined functional imaging techniques to assess response after only one cycle.^3–5^ Amongst the functional imaging techniques ^18^F-FDG PET has been used to assess changes in tumour glucose metabolism following NACT^6^ and a number of PET studies have looked specifically at breast tumour blood flow with either [15-O] H_2_O PET^7^ or dynamic ^18^F-FDG PET.^8^ However, repeated PET imaging is impractical for routine clinical use^9^ and so MRI techniques that measure parameters related to flow, via dynamic contrast-enhanced (DCE) MRI, have also been studied.^10^ A difference between these MRI approaches and PET imaging of blood flow is the lack of absolute quantification by MRI. Moreover, there has been a lack of consistency in study methodology and the reporting of pathological response in MRI studies over the years.^10–12^

In this study, we used a recently developed interleaved high spatial resolution (HSR)/high temporal resolution (HTR) MRI technique^13^ to measure tumour blood flow at baseline and after one cycle of NACT in order to test the hypothesis that the change in blood flow after one cycle of NACT would predict pathological response as defined according to international recommendations.^14^ Analysis of the HTR data to estimate blood flow also allowed us to assess related haemodynamic parameters such as blood volume, capillary permeability surface–area product (PS) and interstitial volume and, by using the HSR data, we were able to assess tumour volume.

## Methods and materials

### Patients

In this prospective study consecutive patients, with newly diagnosed primary invasive carcinoma of the breast due to undergo NACT with curative intent, were approached and asked if they were willing to take part. All those agreeing to join the local Research Ethics Committee approved study provided written informed consent after the nature of the procedures had been fully explained. Each underwent a block sequential regimen of NACT: three 3-weekly cycles of EC90 [epirubicin (90 mg/m^2^) and cyclophosphamide (600 mg/m^2^)] followed by three 3-weekly cycles of docetaxel (100 mg/m^2^). In patients with HER2 positive tumours, docetaxel was accompanied by trastuzumab, and in some (more recent) cases pertuzumab. Blood samples, taken as standard of care during clinic visits, were used to estimate haematocrit at each MRI visit.

### MRI

MRI was performed before initiation of NACT and then repeated in the 2-week period immediately before administration of the second cycle of EC90. All imaging was performed using a 1.5 T MR system (Aera, Siemens Medical Systems, Erlangen, Germany) with the patients positioned head-first and prone. A dedicated 16-channel breast coil and, where possible, an additional flexible array coil placed on the patients back to increase the signal from the descending aorta^15^ were employed. The flexible array was omitted in eight of the subjects who were too large for its use or found the additional coil too uncomfortable.

The MRI protocol included localizing images, *T*_1_-, *T*_2_- and diffusion-weighted imaging before a 3D non-selective inversion recovery (IR) prepared spoiled gradient echo sequence (FOV: 340 × 340 × 180 mm, matrix size: 128 × 128 × 36, IR-TR: 3000 ms, TR/TE: 2.8/0.93 ms, flip angle (FA): 8°, GRAPPA parallel factor: 2, acquisition time: 1 min 5 s per inversion time) was used at four inversion times (100, 600, 1200 and 2800 ms) to estimate *T*_1_. The volume selected encompassed both breasts, the aortic arch and part of the descending aorta.^15^ This was followed by interleaved HTR and HSR DCE sequences.^13^ The DCE series consisted of 93 HTR volumes interleaved with 8 HSR volumes (10 × HTR, 1 × HSR, 43 × HTR, [1 × HSR, 5 × HTR] repeated seven times, 5 × HTR). For the HTR dynamic data, a *T*_1_ weighted 3D spoiled gradient echo sequence (FOV: 340 × 340 × 180 mm, matrix size: 128 × 128 × 36, TR/TE: 2.37/0.73 ms, FA: 25°, CAIPIRINHA parallel factor: 2 × 2, acquisition time: 2 s) was employed. The HSR data were acquired using a fat-suppressed *T*_1_ weighted 3D spoiled gradient echo sequence (TR/TE: 4.1/1.2 ms, FA: 10°, GRAPPA parallel factor: 3, matrix size: 384 × 384 × 128, acquisition time: 36 s). Both HTR and HSR acquisitions encompassed the same volume as the IR sequence. Gd-DOTA (Dotarem, Guerbet, Laboratories, Aulnays Sous Bois, France) was administered intravenously via the antecubital vein using an automated power injector (Spectris Solaris EP, Bayer, Leverkusen, Germany), at the beginning of the 11th dynamic volume (dose of 0.1 mmol/kg) followed by 20 ml of saline at a rate of 3 ml/s. After all eight HSR volumes had been acquired (and 88 HTR volumes) a second, bookend, IR *T*_1_ measurement was made,^16^ and then the final five HTR volumes were acquired.

### Data analysis

Where more than one tumour was identified in a patient, analysis was performed on the largest tumour only. Regions of interest were drawn manually on the HSR images to estimate enhancing tumour volume (these regions were drawn on every slice in which the tumour appeared but excluded areas of necrosis or breast marker clips). These were typically drawn on the first post-contrast volume (acquired approximately 90 s after arrival of contrast agent) and subtracted images were used as a visual aid in the outlining. The multislice regions of interest were drawn by one reader (IMF) and checked by a second (NS; an experienced breast radiologist). Matching regions were drawn on the HTR images and these were used to generate signal-time curves as well as to estimate *T*_1_ relaxation times from the IR data. Further regions, drawn in the descending aorta, were used to generate SI-time curves and estimate *T*_1_ before and after contrast agent administration to calculate an arterial input function.^15^ The SI-time curves and bookend *T*_1_ estimates were used, with an iterative scheme, to convert signal to contrast agent concentration.^15,16^ These data were then fitted using locally developed software produced in Matlab (Mathworks; Natick, MA) and a two-compartment exchange model to provide estimates of blood flow, PS, interstitial volume and blood volume.^17^

### Ki-67 and pathological assessment

Each of the patients went on to receive a further two cycles of EC90 and then three cycles of docetaxel, with or without HER2-targeted therapy, before undergoing surgery. The resected surgical specimens were dissected and the histology examined by a specialist pathologist who derived a residual cancer burden (RCB) index.^14^ Patients with an RCB class of 0 or I (RCB index ≤1.36) were deemed to be pathological responders (pR) and those with an RCB class of II or III (RCB index >1.36) were deemed to be pathological non-responders (pNR). In addition, diagnostic biopsy specimens were retrieved and Ki-67 expression was assessed. Four-micron sections were taken onto X-tra microscope slides (Leica, Wetzlar, Germany) and antigen retrieval was performed using Antigen Unmasking Solution (Vector Laboratories, Burlingame, CA) and heat (pressure cooker for 2 min). Endogenous peroxidase was blocked with 0.3% hydrogen peroxide (10 min). Slides were stained with 1:400 dilution of anti Ki-67 antibody (M7240, Dako; Agilent, Santa Clara, CA) for 1 h at room temperature, and staining was visualised using Impress reagents (Vector Laboratories). Ki67 staining was quantified using the Whole Section Scoring Protocol developed by The International Ki67 in Breast Cancer Working Group.^18^ Results were reported as a percentage of tumour nuclei expressing Ki-67.

### Statistical methods

Data were summarised using mean ± standard deviation when they were normally distributed and median [interquartile range] when they were not (assessed using the Shapiro–Wilk test). To determine whether differences existed between the measured parameters, estimates from pR and pNR were compared using the non-parametric Mann–Whitney *U* test and cycles were compared using the Wilcoxon signed rank test. Response prediction was assessed using receiver operating characteristic (ROC) curves. *p* < 0.05 was considered to indicate a statistically significant difference.

## Results

Between August 2015 and April 2018, 40 female patients (median age 45 years, range 25–69 years) gave written informed consent to enter the study. MRI data were obtained in all 40 patients at baseline (7 [4 - 12] days before the first administration of NACT). However, one patient was unable to tolerate the complete imaging protocol and technical problems in one other (back coil was switched on and off intermittently) precluded the measurement of tumour blood flow. Following a single cycle of EC90, 37 patients were scanned (three patients declined further MRI scans). Technical problems (as previously) affected one further scan, so blood flow estimates were obtained in 36 patients. Paired baseline and cycle one data were available for 35 patients and the results presented below reflect these 35 patients only (Table 1).

**Table 1.** Clinical characteristics of the study population

Characteristic	Number
Median age (years)	45 (range, 25–69)
Tumour type	
Invasive ductal carcinoma	33
Invasive (type not specified)	2
Grade	
II	15
III	20
Oestrogen receptor status	
Positive	24
Negative	11
Progesterone receptor status	
Positive	16
Negative	17
Not evaluable	2
HER2 status	
Positive	14
Negative	21
Median Ki-67 expression (%)	45 (range, 4.3–83.3)

The immunohistochemical subtype (IHS) of the 35 tumours were: 2 HER2 enriched (oestrogen receptor and progesterone receptor negative and HER2 positive), 9 triple negative and 24 luminal B (oestrogen receptor and/or progesterone receptor positive and HER2 positive or HER2 negative with high levels of Ki-67). Following the completion of NACT and surgery, pathological analysis revealed 6 patients (17%) had a pathological complete response (RCB-0), 9 patients had minimal residual disease (RCB-I), 12 patients moderate (RCB-II) and 8 patients extensive residual disease (RCB-III). Thus, there were deemed to be 15 pR (43%) and 20 pNR (57%).

Pathological complete response to NACT varied as a function of IHS – neither of the patients with HER2 enriched tumours, 3/9 (33%) patients with triple negative tumours and 3/24 (12.5%) patients with luminal B tumours had a complete pathological response. Less variation was seen in pR where 1/2 (50%) patients with HER2 enriched tumours showed pR compared to 3/9 (33%) with triple negative tumours and 11/24 (46%) with luminal B tumours. Luminal B tumours that were HER2 positive responded better to NACT: 9/12 (75%) HER2 positive tumours had a pR compared to only 2/12 (17%) HER2 negative tumours. Median enhancing tumour volume at baseline was 4.8 [2.3 to 22.5] cm^3^ and patients going on to a pR had tumours that were significantly smaller than those that went on to pNR (*p* = 0.003). There was a small but significant decrease in tumour volume following one cycle of NACT (median volume after one cycle 5.3 [1.6 to 14.4] cm^3^, median decrease in volume after one cycle 0.48 [0.0 to 5.03] cm^3^; *p* = 0.005) and tumour volume at baseline and after cycle one were good predictors of pR (area under the ROC curve 0.80 (95% CI 0.66 to 0.95) and 0.81 (95% CI 0.66 to 0.95), respectively). Using a volume threshold at cycle one to predict response failure, 12 patients with tumours of 9 cm^3^ or larger went on to pNR (20 patients scanned after cycle one went on to pNR; the test had a sensitivity of 60%) and all 15 patients who went on to a pR had tumours smaller than 9 cm^3^ (100% specificity). This test had a positive predictive value of 100%.

Tumour blood flow at baseline (0.32 ± 0.17 ml/min/ml) and after cycle one (0.28 ± 0.18 ml/min/ml) showed little variation as a function of IHS (Table 2) or pathological response. PS (0.06 ± 0.04 ml/min/ml at baseline, 0.05 ± 0.03 ml/min/ml after cycle one), blood volume (0.33 ± 0.15 at baseline, 0.34 ± 0.13 after cycle one) and interstitial volume (0.22 ± 0.08 at baseline, 0.23 ± 0.08 after cycle one) similarly showed no significant variation with IHS or pathological response. Tumour *T*_1_ at baseline (1287 ± 87 ms) showed no change following one cycle of NACT (1285 ± 92 ms) and showed no significant variation as a function of IHS (Table 2).

**Table 2.** Results of the *T*_1_ & DCE analysis at baseline as a function of tumour immunohistochemical subtype

	Triple negative	Luminal B	HER2 enriched
	(*n* = 9)	(*n* = 24)	(*n* = 2)
Tumour volume (cm^3^) median (IQ range)	10.7 (4.4–23.6)	4.1 (1.8–24.0)	6.9 (4.6, 9.2)^*a*^
Blood flow (ml/min/ml tissue)	0.31 ± 0.12	0.31 ± 0.18	0.57 (0.52, 0.62)^*a*^
Blood volume (no units)	0.31 ± 0.14	0.35 ± 0.16	0.25 (0.17, 0.32)^*a*^
PS (ml/min/ml tissue)	0.08 ± 0.03	0.05 ± 0.03	0.12 (0.09, 0.14)^*a*^
Interstitial volume (no units)	0.22 ± 0.07	0.21 ± 0.08	0.26 (0.19, 0.32)^*a*^
Tumour *T*_1_ (ms)	1315 ± 72	1272 ± 92	1345 (1336, 1354)^*a*^

a mean (both raw values reported).

Patient haematocrit decreased from a baseline value of 0.41 ± 0.03 to 0.39 ± 0.03 (*p* = 0.007) after one cycle of NACT and this was mirrored in an increase of blood *T*_1_ from a baseline value of 1660 ± 64 ms to 1766 ± 109 ms (*p* = 0.00002) after one cycle of NACT.^15^

## Discussion

**Figure 1.**
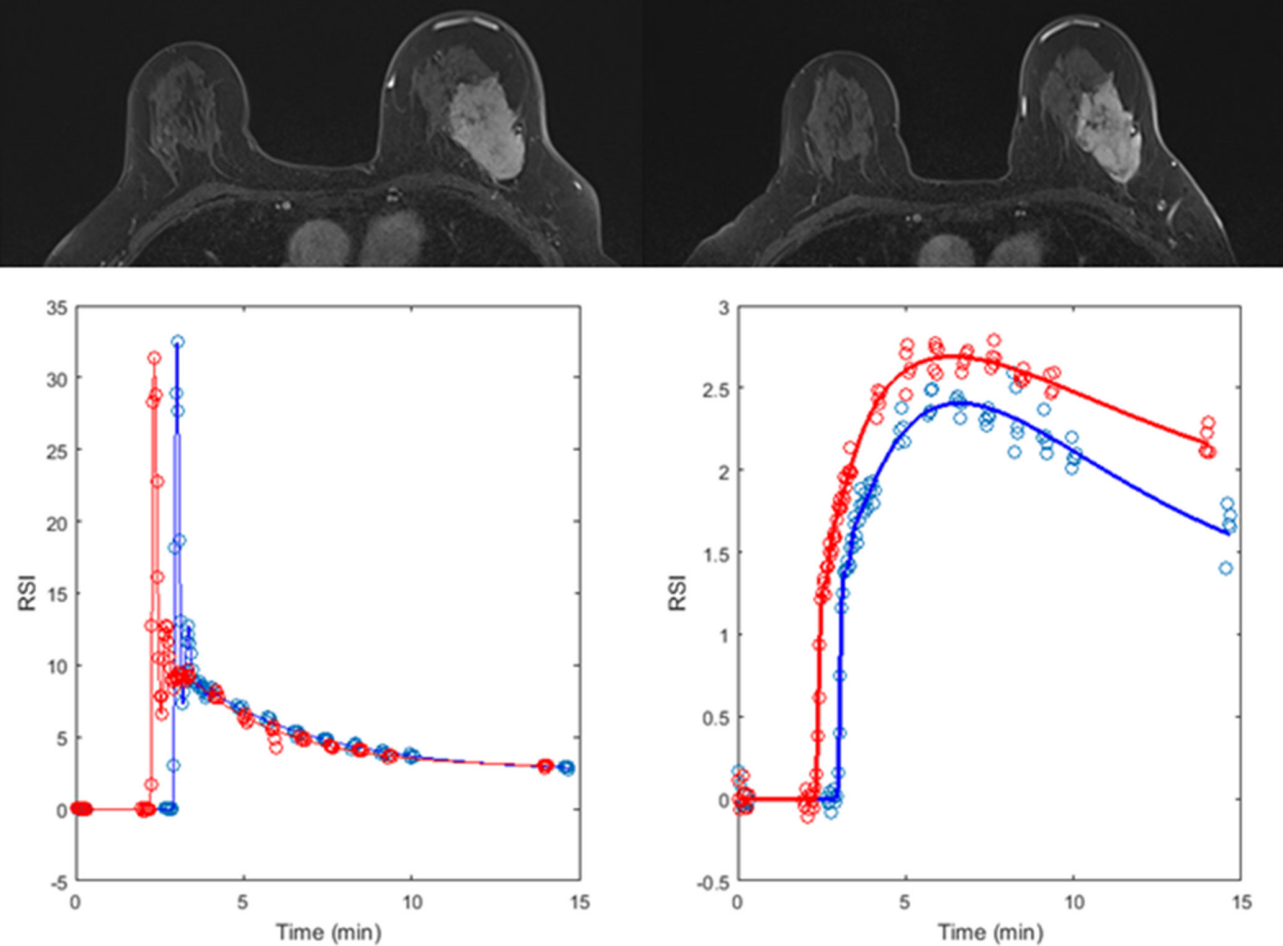
A 51-year-old female with a Grade 3 luminal B invasive ductal carcinoma in the left breast. *T*_1_ weighted HSR images (top) highlight a tumour at baseline (left) with a volume of 45 cm^3^ which reduced to 32 cm^3^ after one cycle of NACT (right). Analysis of the DCE-MRI data using arterial input functions measured in the descending aorta (bottom left, relative change in signal intensity *vs* time) produced estimates of blood flow of 0.24 ml/min/ml tissue at baseline (blue data points and curves) and 0.26 ml/min/ml tissue after one cycle (red data points and curves, bottom right). Following completion of NACT, the patient underwent a left mastectomy and pathological assessment of the resected specimen revealed an RCB index of 3.4 (RCB class III, pNR).

**Figure 2.**
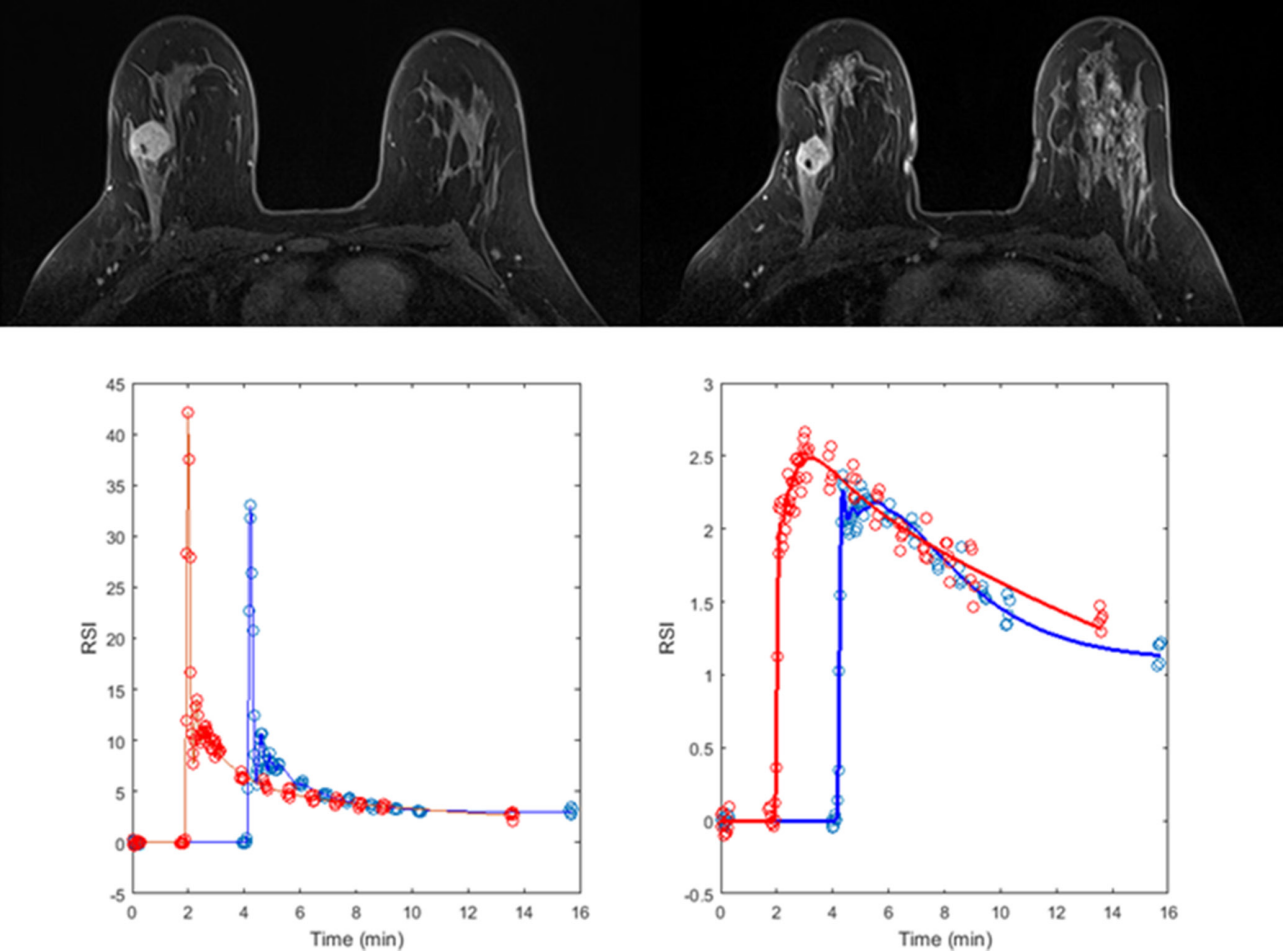
A 37-year-old female with a Grade 3 triple negative invasive ductal carcinoma in the right breast. *T*_1_ weighted HSR images (top) highlight a tumour at baseline (left) with a volume of 3.1 cm^3^ which reduced to 2.9 cm^3^ after one cycle of NACT (right). Analysis of the DCE-MRI data using arterial input functions measured in the descending aorta (bottom left) produced estimates of blood flow of 0.46 ml/min/ml tissue at baseline (blue data points and curves) and 0.31 ml/min/ml tissue after one cycle (red data points and curves, bottom right). Following completion of NACT, the patient underwent a wide local excision and pathological assessment of the resected specimen revealed an RCB index of 0 (RCB class 0, pR).

At least one previous [15-O] H_2_O PET study reported a large differential response of tumour blood flow in groups of patients who had pR and pNR, though these data were acquired after 9 weeks (multiple cycles) of NACT.^7^ We did not see this effect in our study following just one cycle of NACT. There was, on average, a decrease in blood flow of 12% but this was subject to considerable variation, with both increases and decreases seen in both pR and pNR (Figures 1 and 2). Our results reflect those of Humbert et al^8^ who saw a large variability in blood flow response in HER2 negative tumours after one cycle of EC chemotherapy (HER2 positive patients in that study experienced a large decrease in blood flow but they were selectively treated with docetaxel and trastuzumab). The primary hypothesis of this study - that change in blood flow after one cycle would predict pathological response - was not supported by our results

Conversely, tumour volume, which was identified as a more reliable marker of response than tumour diameter in a previous review,^10^ was predictive of response. This mirrors results seen in the ACRIN 6657 trial that highlighted the importance of enhancing tumour volume, measured at cycle one, to recurrence-free survival.^19^ At cycle one of our study, 12 of the 35 tumours were 9 cm^3^ or larger (7 of these tumours were luminal B, 4 were triple negative and 1 was HER2 enriched). These patients could potentially have been spared further NACT since all failed to respond (pNR). Of the remaining 23 patients with tumours smaller than 9 cm^3^, 15 went on to a pR leaving only 8 non-responding patients. Further work is required to identify a specific imaging phenotype to identify those tumours at the early stages of NACT.

This is the first published study to report absolute estimates of breast tumour blood flow and related parameters measured using MRI in response to treatment.^20^ Previous quantitative MRI studies have typically reported parameters such as K^trans^ (volume transfer constant) without measuring patient-specific arterial input functions^21–23^ or by measuring the input function inadequately^24^ (compare the input functions presented in figures 2 and 3 of Tateishi et al^24^ with those presented in Georgiou et al^15^ obtained following very similar injection protocols). It is well recognised that measurement of the arterial input function is very challenging.^11^ As in the current study, some reports have highlighted the importance of tumour size after a single cycle of NACT as a predictor of response while tracer kinetic parameters have been less useful^21,25^; others found neither size nor tracer kinetic parameters useful at this timepoint.^22^ Although the current study lacks direct validation of the blood flow estimates, the values reported very closely reflect previously published estimates obtained using the ‘gold-standard’ of [O-15] H_2_O PET in a similar group of patients.^26^ Moreover, the lack of variation in tumour blood flow as a function of IHS seen in the PET study^26^ is similarly seen in our data (at least in luminal B and triple negative cancers). Despite the well-established variation in response to NACT of the different IHS of breast cancer, there appears to be very little difference in their haemodynamic characteristics at baseline and no clear patterns of change following one cycle of NACT. The decrease of blood flow seen in responders later in the course of NACT by others^7^ is not yet apparent after one cycle. Measurements of blood flow may prove to be more revealing when assessing NACT regimens with expected antiangiogenic effects.^8^

### Limitations

The additional time required to acquire data for blood flow estimation amounts to less than 10 min (bookend *T*_1_ measurements plus a few additional DCE acquisitions) and this could be reduced if a faster B1-insensitive *T*_1_ measurement sequence were employed. The interleaving of HTR data with the clinical HSR data did not have a significant impact on clinical reporting.^13^ Perhaps more significant limitations are the small number of patients studied and the post-processing overhead both for tumour volume estimation and tracer kinetic analysis, which currently rely on in-house software. Further work is required to develop user-friendly tailored software for these applications.

## Conclusions

This study has reported, for the first time, absolute estimates of tumour blood flow and related haemodynamic parameters measured using MRI in a cohort of patients with breast cancer undergoing NACT. Those parameters (blood flow, PS, blood volume, interstitial volume) show little variation with tumour IHS at baseline or in response to one cycle of NACT and, contradicting our primary hypothesis, blood flow change following one cycle of NACT did not predict pathological response. In contrast and reflecting the findings of a recent multicentre trial, enhancing tumour volume measured after one cycle of NACT appears to show promise for prediction of pathological response.
